# Hydrodynamic rupture of liver in combat patient: a case of successful application of “damage control” tactic in area of the hybrid war in East Ukraine

**DOI:** 10.1186/s40792-017-0363-6

**Published:** 2017-08-15

**Authors:** Igor Khomenko, Vitalii Shapovalov, Ievgen Tsema, Georgii Makarov, Roman Palytsia, Ievgen Zavodovskyi, Ivan Ishchenko, Andrii Dinets, Vladimir Mishalov

**Affiliations:** 1 Department of Abdominal Surgery, National Military Medical Clinical Center of Ministry of Defense of Ukraine, Kyiv, Ukraine; 2 Military Medical Clinical Center of South Region of Ministry of Defense of Ukraine, Odesa, Ukraine; 3 Department of Surgery #4, Bogomolets National Medical University, Olexandrivska Teaching Hospital, Shovkovychna 39/1 str., Kyiv, 01601 Ukraine

**Keywords:** Thoraco-abdominal combat injury, Hydrodynamic liver rupture, Damage control tactic

## Abstract

**Background:**

The hybrid war of Russia against Ukraine has been started in certain districts of Donetsk and Luhansk oblasts within the Donbas area in 2014.

**Case presentation:**

We report a clinical case of a combat patient who was injured after the multiple launcher rocket system “Grad” shelling, diagnosed with hydrodynamic liver rupture followed by medical management with application of damage control (DC) tactic in conditions of hybrid war.

The patient underwent relaparatomy, liver resection, endoscopic papillosphincterotomy, endoscopic retrograde cholecystopancreatography, stenting of the common bile duct, and VAC-therapy. Applied treatment modalities were effective; the patient was discharged on the 49th day after injury.

**Conclusions:**

To our best knowledge, this is the first report describing a successful application of DC tactic in the hybrid war in East Ukraine. From this case, we suggest that application of DC tactic at all levels of combat medical care could save more lives.

## Background

The hybrid war of Russia against Ukraine has been started in certain districts of Donetsk and Luhansk oblasts within the Donbas area in 2014. Although the actual nature of this armed conflict is a war, it is officially entitled by the Ukrainian government as an antiterroristic operation or ATO [1, 2]. The pro-Russian terroristic forces apply modern weapons such as multiple-launch rocket systems (MLRS) “Grad”, “Smerch”, or “Uragan” against the Armed Forces of Ukraine during the hybrid war. It has resulted in various injuries of military personnel, which is not typically seen in known armed conflicts [3–6]. Out of all injuries, thoraco-abdominal fragmental wounds are considered as the most severe [3, 7, 8]. First aid is provided during the evacuation or at the place of injury (I level), whereas hospital stage of medical care in Ukraine to the combat patients is organized at the levels II, III, IV, and V [7, 9]. The surgical care (II level) is provided in district hospitals to be deployed very close to the battlefield line, contributing to the implementation the principle of the “golden hour”.

Specialized surgical care (level III) is provided in Kharkiv and Dnipro. The IV level of medical care is provided in national (Kyiv) or regional Military Medical Clinic Centers (Vinnytsya, Odesa, Lviv), in which specialized surgical aid and high-tech medical equipment are available.

Damage control (DC) is a clinical approach in combat conditions to be applied in the US army and military forces of NATO, and in some other armies to improve the management of combat patients [7, 10]. In brief, a DC is a combination of DC surgery and DC resuscitation aiming to control hemorrhage and microbial contamination followed by intraperitoneal or thoracic packing and rapid closure of wounds, resuscitations to correct fluid homeostasis, to control coagulopathy in the intensive care unit (ICU) as well as application of re-exploration [7, 11, 12].

The experience of medical departments of the US army and NATO is considered as modern, and its application to military personnel wounded in the hybrid war in East Ukraine is reasonable. Furthermore, DC is not frequently applied in Ukraine and little is known about consequences of MLRS application to military personnel.

We report a clinical case of a combat patient who was injured after the MLRS “Grad” shelling, diagnosed with hydrodynamic liver rupture and treated with application of DC tactic.

### Ethics, consent, and permissions

The study was approved by the ethical committee at Bogomolets National Medical University (07/10/2015 Nr.90), and written informed consent to participate in the study was obtained. The manuscript does not contain any individual person’s data.

## Case presentation

**Fig. 1:**
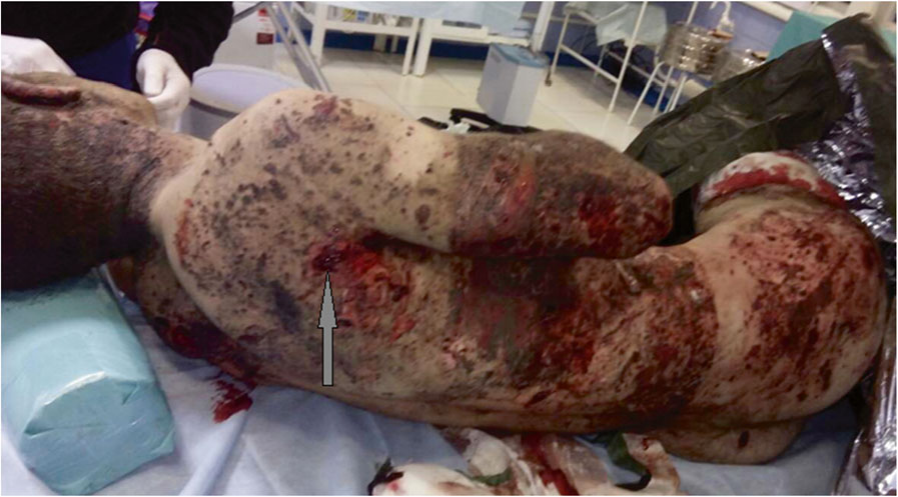
Presentation of the combat patient after being injured by multiple-launch rocket system “Grad” (first hours after injury). The entrance wound from the shrapnel channel is marked by the arrow

The patient was a 22-year-old male soldier who received severe injury in the battlefield area next to the village of Krasnohorivka in the Donetsk oblast of Ukraine. On December 16, 2016, the patient received multifragmental combined with thoraco-abdominal injuries as a result of shelling from MLRS “Grad”. The wounds were detected mainly on the right side of the patient due to fragments penetrating the chest and causing hemopneumothorax (Fig. 1); firearm fracture of 5–7, rupture of right dome of the diaphragm, as well as ruptures of the liver’s parenchyma due to “hydrodynamic shock”. The entrance wound was detected in the region of 5–7 ribs between the anterior and the posterior right axillary lines, whereas an exit wound was not detected.

The patient received first medical aid immediately at the place of injury from other soldiers of his team. This included superimposed aseptic dressings and painkillers. Then, the patient was subsequently evacuated by sanitary transport to the second level of medical care to get primary surgical aid. The time taken between injury and evacuation was less than 60 min; thus, the principle of the “golden hour” was achieved.

To receive basic surgical aid, the patient was transported to the Military Medical Hospital #66 at Pokrovsk of the Donetsk oblast. At this level, the patient was diagnosed with having a penetrating thoracic wound. The blood loss was approximately 1500 ml, indicating hemorrhagic shock. The blood product transfusion of red was applied by using red blood cells and blood plasma.

**Fig. 2:**
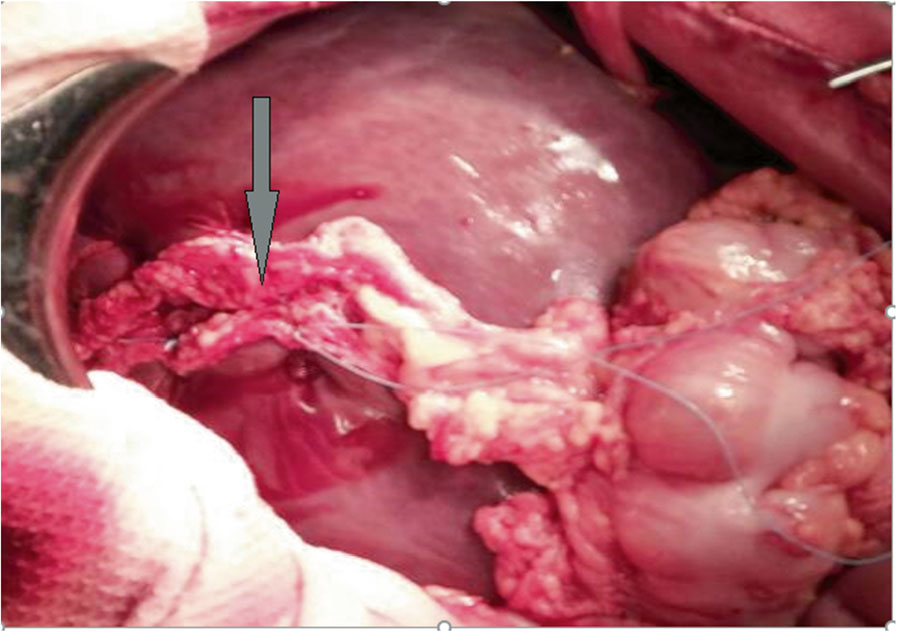
Demonstration of liver damage (second day after injury): damaged tissues were sutured and omentopexy was applied (marked by the arrow)

Thoracentesis was performed to eliminate the right hemopneumothorax. Taking into consideration the absence of an exit wound, it was not possible to be sure about the path of the wound channel; therefore, penetrating thoraco-abdominal injury was suspected and laparocentesis was performed. A hemorrhagic content was received at the laparocentesis; thus, laparotomy was performed. At the revision of the abdominal cavity, we detected a rapture of the right dome of the diaphragm as well as the ruptures of 3–6 segments (S_3–6_) of the liver. The entrance wound was detected only in chest area, whereas the exit wound was not identified neither in chest, nor in abdomen wall. Therefore, a conclusion was made that damage of the liver was in result of “hydrodynamic shock”. Considering the severity of the patient, minimal surgical treatment was applied, which included suturing of liver ruptures as well as excision of necrotic tissues and suturing of gunshot wounds of limbs. However, in an early post-operative period, a hemorrhage and bile were detected on drainage. Relaparotomy was performed and liver damage within the area of gallbladder fossa was detected. Given the doubts about the viability of the liver S_3–6_, the question was whether or not liver resection should be applied for possibly ischemic segments. However, the severity of the patient and principles of DC tactic were taken into consideration. Thus, cholecystectomy was performed to get access to damaged liver in the area of gallbladder fossa (coagulation and suturing), and patch of omentum was used to fill the gap in injured S_3–6_ of liver (Fig. 2).

For further treatment, the patient was transported to the Mechnikov Regional Clinical Hospital in Dnipro, where further resuscitation was continued. Antibacterial therapy was administrated as well as other symptomatic therapy and daily dressings were applied.

Three days after the level III, the patient was transported to the ICU at the National Military Medical Clinical Center of Ukraine in Kyiv. The general condition of the patient remained severe. Drainage from the subhepatic area showed daily bile volume up to 300 ml. Such a volume of bile was caused by the intrahepatic biliary hypertension as a result of post-traumatic edema of liver parenchyma. In order to eliminate biliary hypertension (sixth day after the combat injury), the following intervention was performed: endoscopic papillosphincterotomy, endoscopic retrograde cholecystopancreatography, and stenting of the common bile duct. To provide enteral nutrition, the feeding tube was placed behind the Treitz ligament. Implementation of endoscopic decompression of the bile duct had resulted in gradual reduction of bile volume from the subhepatic drainage from up to 50 ml per day as compared to 300 ml before the decompression.

**Fig. 3:**
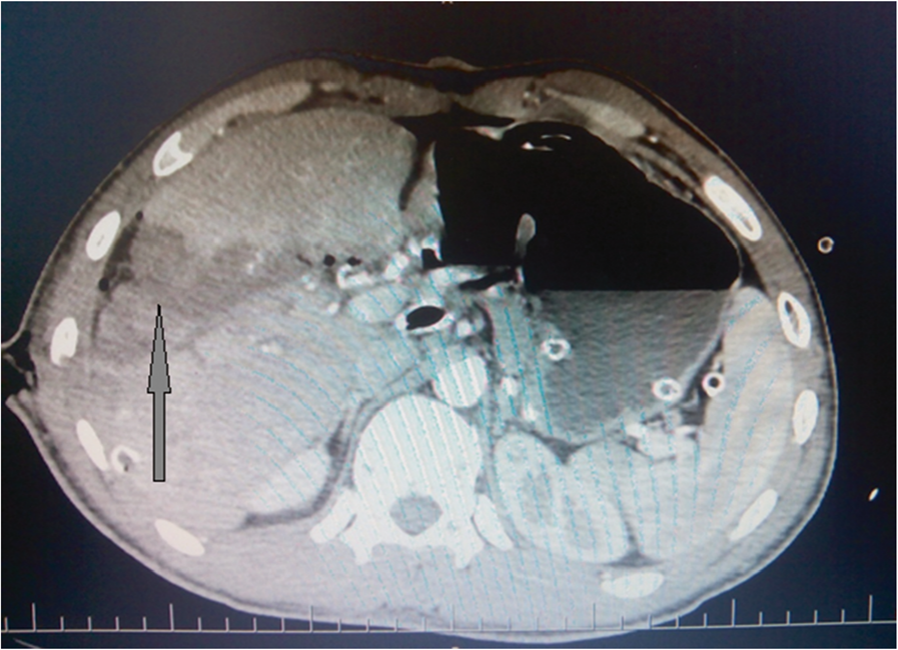
Illustration of abdominal CT scan on the 14th day after injury. The zone of the irreversible ischemia of the fifth to sixth segments of the liver is marked by arrow

**Fig. 4:**
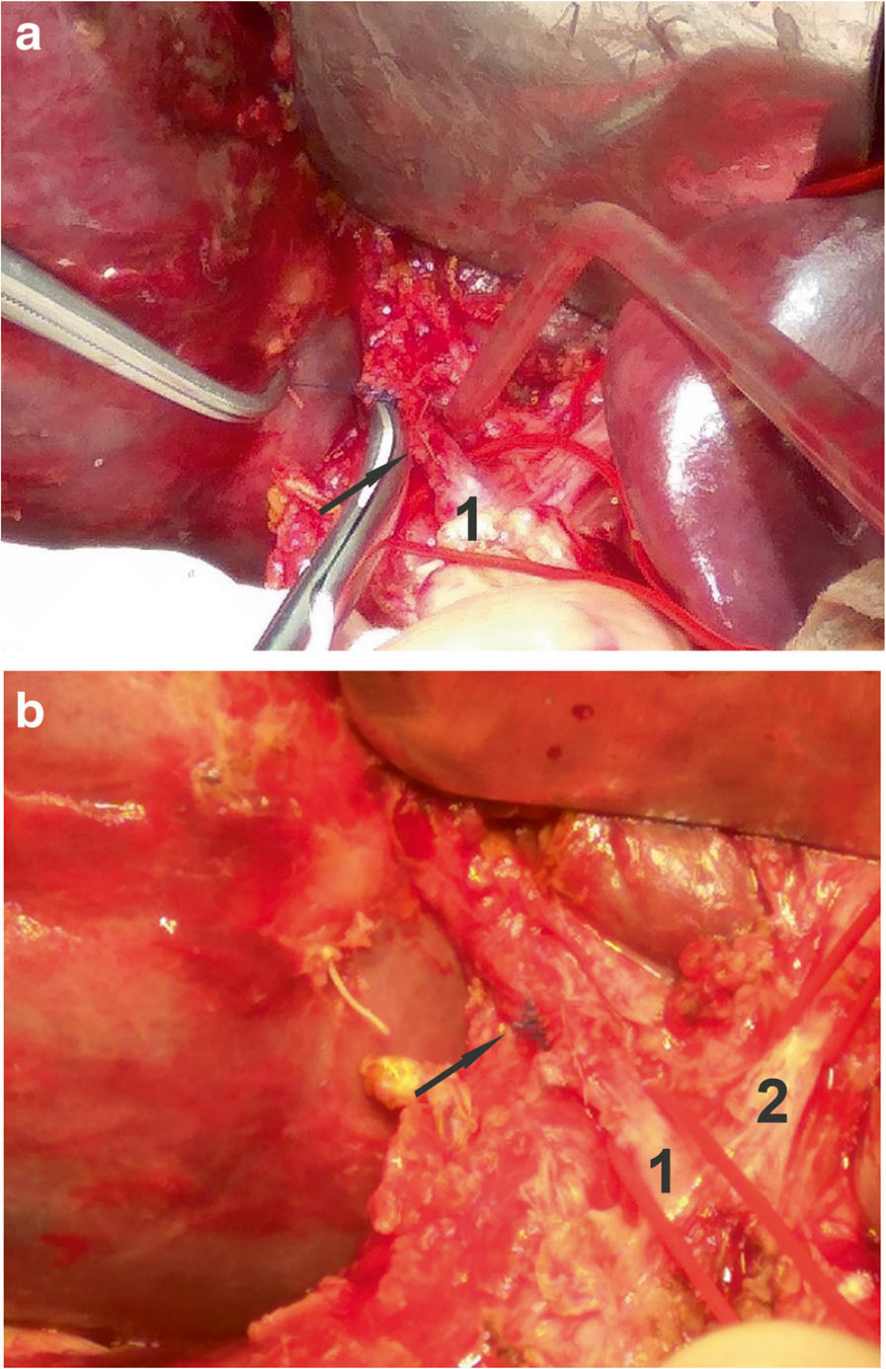
Photograph illustrating erosive defect of right hepatic artery after suturing. **a** The area of erosive defect of right hepatic artery is marked by an arrow; the turnstile is placed under the artery (marked by 1). **b** Sutured right hepatic artery (suture line marked by the arrow): 1 - right hepatic artery; 2 - left hepatic artery on the turnstile

**Fig. 5:**
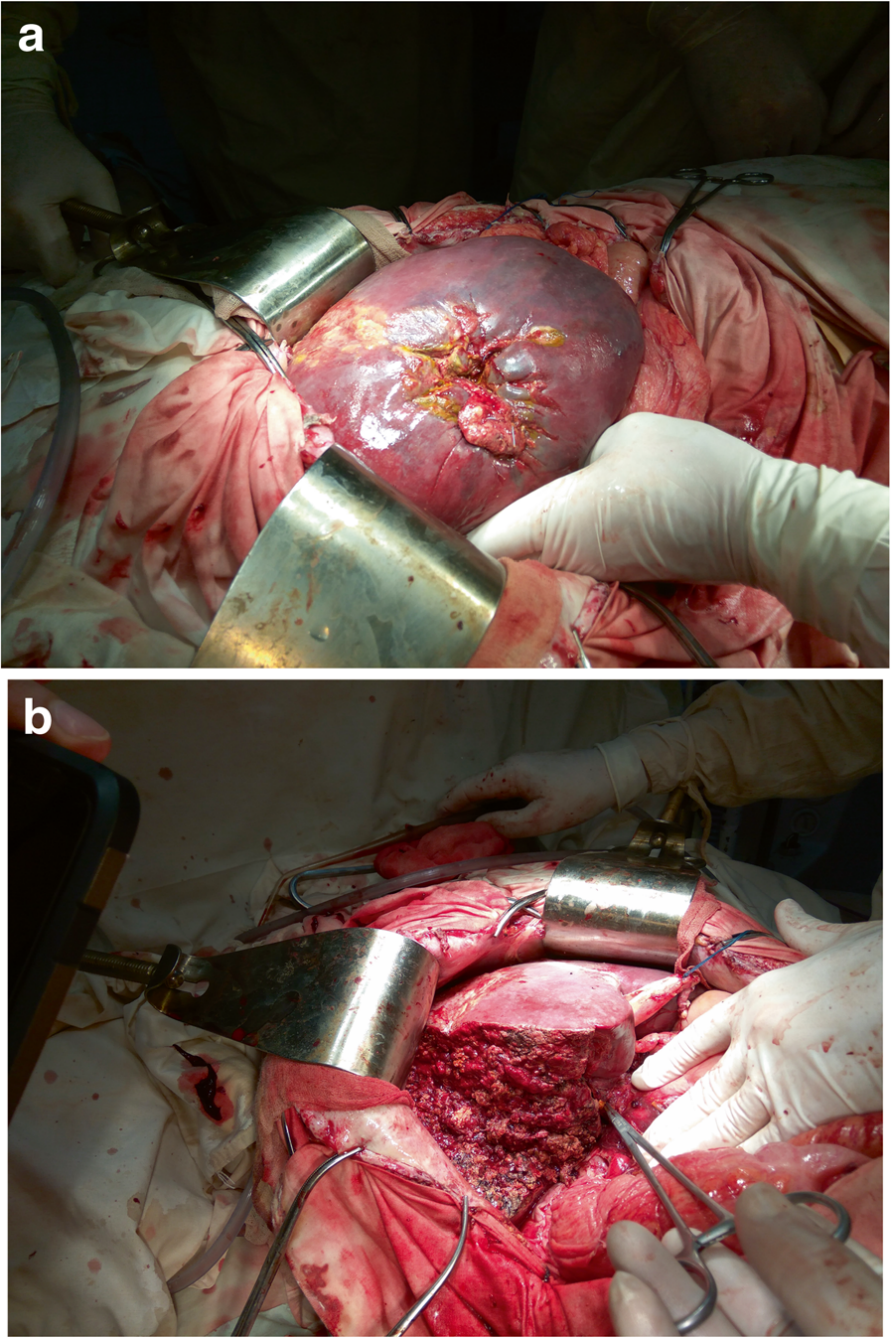
Intraoperative photograph illustrating liver at relaparatomy on the 16th day after the injury. **a** Damaged area of liver is covered with fibrin, bile leakage is seen under the partial failure of sutures. **b** Liver after the resection of fifth and sixth segments

At post-operative period, a computed tomography (CT) scan of the abdomen was performed. At CT analyses showed the liver to demonstrate zones with ischemic lesions (Fig. 3). Considering liver CT data, a liver resection of the affected segment was planned to prevent further necrosis and abscess formation according to DC tactic. However, on the 16th day after the injury, intra-abdominal bleeding was diagnosed and urgent laparotomy was performed. At revision, erosion of the right hepatic artery was detected (Fig. 4a) as a result of the chronic inflammation of the artery wall and continuous contact to the bile. Erosive defect was sutured and stable hemostasis was achieved (Fig. 4b). At revision of sutured areas of the liver, we found it to be covered with fibrin, partial failure of sutures and bile leakage were observed. Furthermore, tissue softening and color changes of liver S_5–6_ parenchyma were detected, indicating ischemic failure (Fig. 5a). In order to stop bile leakage and to prevent liver failure, a decision was made to perform anatomic resection of the liver S_5–6_ (Fig. 5b). Due to the critical status of the patient and considering DC approach, we postponed the surgery for stabilization of hemodynamics by resuscitation and transfusion of one-group packed red blood cells. At the ninth day after the liver resection, the patient was transferred to the surgery ward from ICU.

**Fig. 6:**
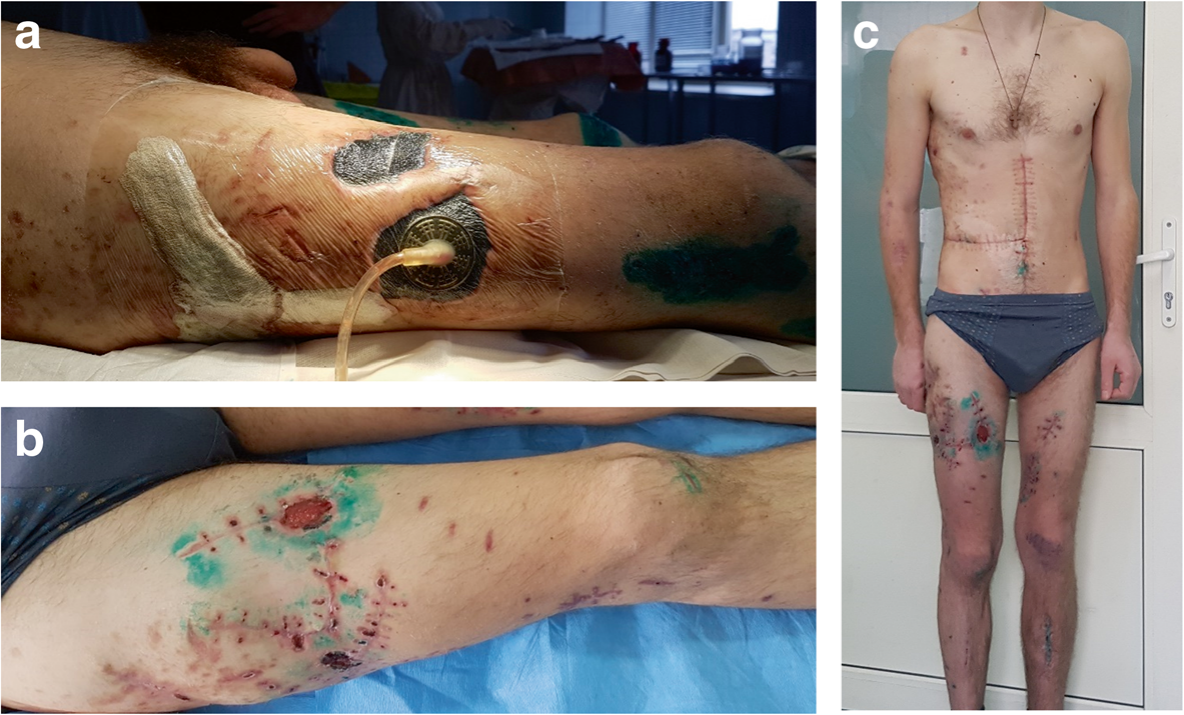
Photograph illustrating the damages of soft tissues after multiple-launch rocket system “Grad”. **a** The shrapnel wounds lateral area of the right thigh with installing of VAC system on the 21st day after the injury. **b** The shrapnel wounds at the anterior area of the right thigh on the 49th day after the injury. **c** The overall look of the patient with post-operative and post-injury scars before discharge from the hospital on the 49th day after injury

Apart from the thoraco-abdominal injuries, the patient was also diagnosed with multiple fragmental injuries of soft tissues of the skull, chest, abdomen, as well as extremities. The most serious soft tissue injury was detected at the right lower limb, which was subsequently treated with VAC system (Fig. 6).

The patient was discharged from hospital in good condition on the 49th day after receiving injuries (Fig. 6c). The patient received 45 days of further rehabilitation according to military medical commission decision before continuing his return to military duties (V level).

## Discussion and conclusions

In this study, we reported a successful application of DC for the combat patient who was severely injured in the Donbas area in the hybrid war in East Ukraine, which is in line with other studies of larger cohorts of combat patients [7, 12]. To our best knowledge, this is the first report to describe a case of medical care service in the hybrid war in East Ukraine. The hydrodynamic rupture of the liver is an uncommon lesion to be diagnosed in relation to thoraco-abdominal injuries in combat patients as showed in large cohort studies [3, 7, 13]. These injuries were caused within the battlefield area of ATO by multiple-rocket launcher system “Grad”, resulting in mine-explosive trauma due to simultaneous effect of the several damaging factors such as an air blast wave, a gas-flame composition, as well as primary shrapnel, which is created from the covering of the explosive ammunition of MLRS “Grad”. Another cause of trauma was due to injury by debris to be formed as a result of the blast wave influence to such objects as bricks, gravel, concrete etc., which is also shown in soldiers from operations by the US Army [3, 4, 7]. An acoustic trauma and barotrauma also were considered because of the high-energy blast wave effect to the human body [14].

Furthermore, in this case report we showed that combat injuries to be received from attacks by the MLRS such as “Grad” demonstrate specific features as compared to mine-explosive injuries received from the mortar and tank shelling, the undermining at the tripwire mine, or the hand grenade explosion. The mechanism of combat injury due to mine-explosion is associated with direct action of the air blast wave, which lead to significant anatomical destruction of the body’s parts (usually limbs) as showed in previously published studies of conflicts in Iraq, Afghanistan, and Chechnya [3–5]. However, in hybrid war in the Donbas, Ukrainian military personnel are injured by rocket shelling from MLRS “Grad” and “Smerch”, which are associated with the impact of high-energy damaging factors as compared to other types of lethal weapons, which is demonstrated in our case of patient with hydrodynamic rupture of the liver. It is worth to mention that frequent application of MLRS “Grad” by pro-Russian separatists against Ukrainian soldiers is one the specific features of the hybrid war in East Ukraine. Furthermore, high-energy effect of the MLRS to military personnel or civilians resulted in changes of the structure in combat trauma as compared to military operations for instance in Iraq, Afghanistan, and Chechnya [3–5]. In the battlefield of East Ukraine, we observe a lower number of traumatic limb amputations, but higher frequency of massive traumatic disruptions of various body parts, which are lethal in majority of cases. Still, lives could be saved after MLRS application, which is demonstrated by the present case.

It is worth to mention that high-energy shrapnel is associated with additional explosive and hydrodynamic effects because of their high kinetic potential, which is more typical for bullet gunshot injuries with bullet speeds above 150 m/s. Moreover, hydrodynamic effect is associated with ballistic pressure waves, occurring while high-energy fragments pass through the human body. The hydrodynamic effect is strong, causing significant anatomical destruction of tissues and organs outside of the trajectory of the formed wound channel, and could result even in bone fractures, which is described as hydrostatic shock by Courtney et al. [15]. The hydrodynamic effect is also described in the present study of the combat patient with a severe injury including hydrodynamic liver rupture. In our case, the hydrodynamic effect occurred when the shrapnel (or caused by shrapnel, the side shock waves) with high kinetic energy penetrated the liver, which is a fluid-filled organ. In our case, hydrostatic effect caused the injury of the right dome of the diaphragm and rupture of the liver S_3–6_ without shrapnel channel in the abdomen. Moreover, the entrance wound was detected in the region of 5–7 ribs between the anterior and the posterior right axillary lines, whereas an exit wound was not detected indicating unknown path of the shrapnel and possible damage of abdominal organs [16]. Not surprisingly, ruptures of S_3–6_ segments of the liver were diagnosed at laparotomy. However, shrapnel in the abdominal cavity was not identified, indicating different mechanism of the liver damage such as hydrodynamic shock [15].

Given the described clinical case and specific features, high-energy wounds from rocket launchers shelling, we consider that it is necessary for all patients with penetrating thorax wounds to perform both thoracocentesis and laparocentesis in order to identify and manage possible thoraco-abdominal injury. For these patients, a CT scan is highly informative; however, it is not available within the “golden hour” period within ATO area in Ukraine. Similar to Kotwal et al., we followed the principle of “golden hour”, aiming to reduce the time between injury and medical care to increase the rate of saved lives of combat patients and in agreement with [17].

For our patient, the DC tactic was applied, which is a highly effective medical approach for management of combat patients. According to DC, we delayed the resection of liver S_3–6_ for 16 days because of hydrodynamic ruptures, which is in line with other studies [7, 8, 10, 12]. According to DC, a critical status of the patient was considered and performing of surgery for patients could be fatal. By using DC, a more careful assessing of the viability of the liver ischemic areas was performed and liver resection with maximum preservation of viable liver parenchyma was possible [6]. Hence, out of four liver segments to be planned for resection at the first laparotomy, two liver segments were preserved because of DC application, and recovering of liver viability through the collateral circulation during 16 days after injury was achieved. This report is a demonstration that patients with severe liver injuries associated with critical health condition after MLRS shelling should undergo DC tactic in condition of hybrid war.

In the presented clinical case, we also showed that suturing of the liver ruptures with omentopexy was not effective and resulted in partial failure of hepatic sutures and bile leakage. Such complication could be avoided if appropriate conditions for adequate drainage of the place of potential sutures failure was considered at the first place to prevent the influence of the bile to injured organs. Furthermore, perihepatic packing could also be considered along with the DC approach [8]. In the current case, the bile outflowing first was noticed from the drainage from the pleural cavity whereas the drainage from subhepatic space was not functional, probably due to the location of the wounds within diaphragmatic surface of the liver, which were directly adjoined to the place of diaphragm rupture. Considering the severe condition of the patient and high operative risk of reoperation, we performed the endoscopic decompression of the bile ducts and to postpone resection of liver, which is in agreement with other studies applying DC control [7, 8, 10, 12]. Unfortunately, we were not able to fully use this time for adequate preparation of the patient to the liver resection because of the bleeding from the erosive defect of the right hepatic artery to be diagnosed at the tenth day after the endoscopic decompression. Thus, endoscopic transpapillary decompression of the biliary tracts was effective in condition of the suture failures.

To summarize, we demonstrated the case of a combat patient with severe penetrating wounds after MLRS shelling from the hybrid war in East Ukraine. To our best knowledge, this is the first report from the Donbas battlefield describing a successful application of DC approach. From this case, we hypothesized that application of DC tactic at all levels of combat medical care could save more lives. Effect of high-energy from MLRS “Grad” could result in liver damage due to hydrodynamic impact of the shelling.

## Abbreviations

ATO: Antiterroristic operation
CT: Computed tomography
DC: Damage control
ICU: Intensive care unit
MLRS: Multiple-launch rocket systems
